# KDM6B inhibition modulates monocyte activation and alleviates IMQ-induced psoriasis-like skin inflammation

**DOI:** 10.1186/s12964-026-03220-4

**Published:** 2026-09-11

**Authors:** Aman Damara, Najla Abassi, Delia Mihoc, Mahsa Nastaranpour, Pauline Kraft, Tina Sarkar, Tanja Kübelbeck, Daniela Kramer, Carsten Deppermann, Johannes U. Mayer, Stephan Grabbe, Michael Delacher, Federico Marini, Fatemeh Shahneh

**Affiliations:** 1 Department of Dermatology, University Medical Center, Johannes Gutenberg-University, Mainz, Germany; 2 Institute of Medical Biostatistics, Epidemiology and Informatics (IMBEI), University Medical Center, Johannes Gutenberg-University, Mainz, Germany; 3 Institute of Immunology, University Medical Center, Mainz, Germany; 4 Center for Thrombosis and Hemostasis (CTH), University Medical Center, Johannes Gutenberg-University, Mainz, Germany; 5 Research Center for Immunotherapy, University Medical Center, Johannes Gutenberg-University, Mainz, Germany; 6 Institute for Molecular Biology, Johannes Gutenberg-University, Mainz, Germany

**Keywords:** Psoriasis, Metabolic modifications, Epigenetic modifications

## Abstract

**Background:**

Inflammatory monocytes are increasingly recognized as key amplifiers of psoriasis, yet the epigenetic drivers of their pathogenic signature remain unclear. The mechanisms linking epigenetic regulation to metabolic reprogramming in monocyte-driven inflammation in psoriasis are poorly defined.

**Methods:**

We examined the expression and activity of the histone demethylase KDM6B in classical monocytes in the imiquimod (IMQ)-induced psoriasis model. Epigenetic changes were assessed by measuring H3K27me3 levels at inflammatory and metabolic gene promoters. Pharmacological inhibition of KDM6-family H3K27 demethylase activity after disease onset was performed using GSK-J4, a cell-permeable prodrug that is intracellularly converted to the active inhibitor GSK-J1. In parallel, inflammatory and metabolic transcriptional programs, bioenergetic profiles, immune cell recruitment to inflamed skin, and single-cell transcriptomic changes in myeloid populations were analyzed.

**Results:**

KDM6B expression and KDM6A/B demethylase activity were increased in classical monocytes during IMQ-induced psoriasis-like inflammation. This was associated with reduced levels of the repressive histone mark H3K27me3, an epigenetic modification linked to chromatin compaction and transcriptional silencing, at the *Il1b*, *Tnf, Pgam1*, *Pgk1*, and *Aldoa* promoters, together with an enhanced inflammatory and glycolytic gene signature. GSK-J4 treatment after disease onset restored H3K27me3 at inflammatory and metabolic loci, suppressed *Il1b* and *Tnf* transcription, normalized bioenergetic profiles, and reduced monocyte and neutrophil recruitment to inflamed skin. Single-cell transcriptomic profiling further revealed that GSK-J4 treatment was associated with reduced cytokine-mediated signaling, glycolysis, and chemotaxis pathways in monocytes, while enriching antigen presentation modules, consistent with a shift toward a homeostatic, antigen-presenting surveillance program in myeloid cells and a regulatory T cell (Treg)-supportive milieu.

**Conclusions:**

Collectively, our data identify KDM6B-associated epigenetic-metabolic remodeling as a component of inflammatory monocyte activation in IMQ-induced psoriasis-like inflammation. GSK-J4 treatment attenuated ongoing inflammation when initiated after disease onset. These findings support further investigation of KDM6-family demethylases as therapeutic targets in psoriasis-like inflammation.

**Supplementary Information:**

The online version contains supplementary material available at https://doi.org/10.1186/s12964-026-03220-4.

## Introduction

Psoriasis is an autoinflammatory disease characterized by keratinocyte hyperproliferation, aberrant IL-23/IL-17 signaling, and systemic inflammation, affecting approximately 0.1–3% of the global population, with substantial geographic variation [1, 2]. Psoriasis patients are at elevated risk of psoriatic arthritis, cardiometabolic comorbidities, and impaired quality of life [3]. Current biologics that neutralize TNF-α, IL-17A/F, or the IL-23 p19 subunit achieve high levels of disease control in many patients; however, primary or secondary treatment failure remains an important clinical challenge, highlighting the need to define additional disease-sustaining pathways [4].

Emerging evidence implicates inflammatory monocytes as important contributors to psoriasis pathogenesis [5–7]. In the widely used imiquimod (IMQ)-induced psoriasis-like mouse model, classical monocytes are mobilized from the bone marrow (BM) and recruited to inflamed skin, where they can differentiate into inflammatory macrophages and monocyte-derived dendritic cells and contribute to the production of inflammatory mediators, including IL-1β, TNF-α, and IL-6 [5, 8–10]. Thus, monocyte recruitment, differentiation, and inflammatory activation represent important components of psoriasis-like inflammation and potential targets for therapeutic intervention.

Monocyte phenotypes and function are tightly controlled by transcriptional reprogramming. Epigenetic modifications, such as changes in histone methylation, and metabolic alterations, including enhanced glycolysis, can cooperatively shape monocyte inflammatory responses [11, 12]. Specifically, the chromatin-modifying enzyme KDM6B, a histone demethylase, removes methyl groups from histone H3K27, reducing the repressive effect of H3K27me3 and facilitating transcriptional activation at selected inflammation-associated loci [13–16]. Elevated KDM6A/B activity has been shown in monocytes isolated from psoriasis patients [16, 17], as well as from individuals with other autoimmune conditions such as rheumatoid arthritis and systemic lupus erythematosus [18, 19]. We previously identified increased KDM6A/B activity in monocytes from patients with psoriasis and linked this activity to metabolic remodeling characterized by enhanced glycolysis and inflammatory activation [17]. Yet, whether this epigenetic-metabolic switch functions in monocytes in the IMQ-induced psoriasis model and whether its inhibition can reverse established disease remains unresolved.

Here, we investigated the contribution of monocytes and KDM6-associated chromatin remodeling to psoriasis-like inflammation using complementary IMQ- and IL-36α/IL-17A-driven mouse models, together with monocyte depletion, pharmacological KDM6B inhibition, single-cell RNA sequencing, chromatin immunoprecipitation, and metabolic flux analyses. Monocyte depletion attenuated local and systemic inflammation in IMQ-treated mice, while inflammatory monocytes exhibited increased KDM6B expression and KDM6A/B activity. GSK-J4 treatment after disease onset reduced psoriasis-like inflammation and inflammatory myeloid-cell accumulation and remodeled transcriptional programs in classical monocytes and Treg cells. At the molecular level, GSK-J4 treatment increased H3K27me3 occupancy at regulatory regions of the *Il1b* and *Tnf* genes and reduced their expression. Furthermore, IMQ-induced inflammation was associated with enhanced glycolytic and mitochondrial activity in BM monocytes and reduced H3K27me3 occupancy at selected glycolysis-associated loci including *Pgam1*, *Pgk1*, and *Aldoa*, whereas GSK-J4 attenuated these metabolic and epigenetic changes.

Collectively, our findings identify KDM6-associated epigenetic-metabolic remodeling as a component of inflammatory monocyte activation in psoriasis-like inflammation and support further investigation of this pathway as a potential therapeutic target.

## Materials and methods

### Psoriasis-like inflammation model

All mouse experiments were conducted in accordance with the relevant guidelines and regulations for animal welfare issued by the Federal State of Rhineland-Palatinate State Investigation Office (Germany). The study was approved under authorization number G22-1–096, and all efforts were made to minimize animal suffering. Wild-type C57BL/6 J mice (both male and female, 20 ± 2 g body weight) were obtained from Jackson Laboratory. Mice were 8–10 weeks old at the start of experiments and housed under specific pathogen-free (SPF) conditions, with controlled temperature and humidity, a 12-h light/dark cycle, and ad libitum access to food and water. Psoriasis-like skin inflammation was induced using imiquimod (IMQ). Mice received topical applications of 50 mg of 5% IMQ cream (Aldara, Meda, Solna, Sweden) or diluted basis crème (DAC) control (sham) cream daily for five consecutive days on the depilated back skin (3 × 2.5 cm area) and 10 mg of 5% IMQ cream per ear. In the IMQ/GSK-J4 and Sham/GSK-J4 groups, mice received daily intraperitoneal (*i.p*.) injections of GSK-J4 (10 mg/kg body weight; Cat# HY-15648B, MedChemExpress), starting on day 2 after the first IMQ application. Disease severity was assessed daily using a modified Psoriasis Area and Severity Index (PASI), evaluating three parameters: erythema, scaling, and skin thickness. Each was scored independently on a scale of 0–4 (0 = none, 4 = severe), with a cumulative score ranging from 0 to 12. Body weight was monitored daily from the start of treatment, and weight change was calculated relative to day 1.

In the IL-36α/IL-17A-mediated psoriasis model, ears of male C57BL/6 mice (30 ± 2 g body weight) were treated with intradermal (i.d.) injections of 1 μg murine IL-36α (Cat#7059-ML, R&D Systems) and 1 μg murine IL-17A (Cat# 12,340,176, ImmunoTools) or PBS control for 5 consecutive days. In the IL-36α + IL-17A/GSK-J4 and PBS/GSK-J4 groups, mice received daily intraperitoneal (i.p.) injections of GSK-J4 (10 mg/kg body weight), starting on day 2 after the first injection.

On day 6, all mice were euthanized, and samples including spleen, ear, back skin, and blood were collected for further analysis. Spleen weights were recorded to assess systemic inflammation. Back skin samples were fixed in 4% formaldehyde (ROTI Histofix, Carl Roth, Germany) for 24 h, paraffin-embedded, and sectioned (5–6 µm). Sections were stained with hematoxylin and eosin (H&E) and imaged at 10 × magnification using an Olympus BX51 microscope.

### Peripheral monocyte depletion

Mice were injected *i.p*. every day for up to 6 days with a combination of 100 µg of anti-mouse CD115-CSF1R (clone: AFS98, Cat# BE0213, Bio X Cel) and 100 µg anti-mouse Ly6C (clone: Monts 1, Cat# BE0203, Bio X Cel) to deplete the respective cell populations (classical monocytes from BM) or IgG2a isotype control antibodies (clone: 2A3, Cat# BE0089, Bio X Cel), respectively. Depleting injections started at day 0, one day before IMQ treatment. Depletion efficiency was assessed by flow cytometry staining for Ly6C and Ly6G in peripheral blood as shown in Fig. S1A.

### Cytokine quantification and ELISA

To obtain samples for ELISA, the skin samples were homogenized in protein lysis buffer (20 mM TRIS–HCl, pH 7.5; 150 mM NaCl; 1% Triton X-100; 1 mM Na2EDTA; 1 mM EGTA; 1 mM β-glycerophosphate; 2 M urea; and 1 × protease inhibitor cocktail) in gentleMACS™ M Tubes in the Miltenyi program Proteins_01 on the gentleMACS™ Octo Dissociator, incubated for 10 min at 4 °C, and then briefly sonicated for 10 min in a Bioruptor (Diagenode). After sonication, the lysates were centrifuged for 5 min at 3000 rpm 4 °C and the supernatant was transferred to a new tube. Protein concentration was determined with the Qubit Protein Assay, according to the manufacturer's protocol (Cat# Q33211, Thermo Fisher Scientific). Skin cytokine concentrations were normalized to total protein and expressed as pg/mg protein. Plasma and skin concentrations of IL-6 (Cat# 431,301, BioLegend), IL-23 (Cat# 433,704, BioLegend), and IL-17A (Cat# 432,504, BioLegend) were determined using ELISAMAX™ ELISA kits. Assays were performed colorimetrically according to the manufacturer's instructions. Absorbance was detected at 450 nm using a Hidex Sense microplate reader.

### RNA isolation and RT-qPCR

On day 6, monocytes were isolated from mouse BM using the EasySep™ Mouse Monocyte Isolation Kit (Cat# 19861 A, STEMCELL™ Technologies). Total RNA was isolated from monocytes using the PureLink™ RNA Mini Kit (Cat# 12,183,025, Thermo Fisher Scientific) according to the manufacturer's instructions. RNA concentration and purity were quantified using a NanoDrop spectrophotometer (Thermo Fisher Scientific). RNA was reverse-transcribed into cDNA using the High-Capacity cDNA Reverse Transcription Kit (Cat# 4,368,814, Applied Biosystems). Gene expression was quantified by real-time PCR (CFX384, Bio-Rad) using primers and SYBR Green qPCR Master Mix (Universal) (Cat# HY-K0501A, MedChemExpress). Relative mRNA levels were normalized to the housekeeping gene *Actb* and expressed as fold changes using the 2 − ΔΔCt method. Primers used for qPCR analysis were as follows: *Kdm6b*_F, 5′-CCTCGTCCTTCCAGGAGTCA-3′; *Kdm6b*_R, 5′-CTCCTGTAGCTGTGGCTTCC-3′; *Tnf*_F, 5′-CAGGCGGTGCCTATGTCTCA-3′; *Tnf*_R, 5′-GGCTACAGGCTTGTCACTCG-3′; *Il1b*_F, 5′-GCCACCTTTTGACAGTGATGAGA-3′; *Il1b*_R, 5′-GGACAGCCCAGGTCAAAGGT-3′; *Aldoa*_F, 5′-ACATTGCTGAAGCCCAACAT-3′; *Aldoa*_R, 5′-ACAGGAAAGTGACCCCAGTG-3′; *Eno1*_F, 5′-CATGGGGAAGGGTGTCTCAC-3′; *Eno1*_R, 5′-GTGCCGTCCATCTCGATCAT-3′; *Gpi1*_F, 5′-CAGAGACAGCAAAGGAGTGG-3′; *Gpi1*_R, 5′-GTAGACAGGGCGACAAAGTG-3′; *Pgam1*_F, 5′-TCTGTGCAGAAGAGAGCAATCC-3′; *Pgam1*_R, 5′-CTGTCAGACCGCCATAGTGT-3′; *Pgk1*_F, 5′-ATGTCGCTTTCCAACAAGCTG-3′; *Pgk1*_R, 5′-GCTCCATTGTCCAAGCAGAAT-3′; *Pkm2*_F, 5′-TCGCATGCAGCACCTGATT-3′; and *Pkm2*_R, 5′-CCTCGAATAGCTGCAAGTGGTA-3′. Gene expression was normalized to the housekeeping gene *Actb* (*Actb*_F, 5′-AGGAGTACGATGAGTCCGGC-3′; *Actb*_R, 5′-GGTGTAAAACGCAGCTCAGTA-3′) and expressed as fold change using the 2^ − ΔΔCt method.

### Chromatin immunoprecipitation (ChIP)

On day 6, monocytes were isolated from mouse BM using the EasySep™ Mouse Monocyte Isolation Kit (Cat# 19861 A, STEMCELL™ Technologies). Chromatin immunoprecipitation (ChIP) assays were performed using the Pierce Magnetic ChIP Kit (Cat# 26,157, Thermo Fisher Scientific) according to the manufacturer's protocol. Immunoprecipitation was carried out using 1 µg of anti-H3K27me3 antibody (Cat# C15410195, Diagenode). Normal rabbit IgG served as the negative control for immunoprecipitation. Quantitative PCR (qPCR) analysis was performed using primers targeting promoter regions of the *Il1b*, *Tnf*, *Aldoa*, *Eno1*, *Pgam1*, *Pgk1*, and *Pkm2* genes. Primer sequences were as follows: *Il1b*-F, 5′-GCAGGAGTGGGTGGGTGAGT-3′; *Il1b*-R, 5′-CAGTCTGATAATGCCAGGGTGC-3′; *Tnf*-F, 5′-TCCTGATTGGCCCCAGATTG-3′; *Tnf*-R, 5′-TAGTGGCCCTACACCTCTGT-3′; *Aldoa*-F, 5′-TCCAGGACAAATGGGACTAC-3′; *Aldoa*-R, 5′-GGTGGCAGGGCCACAGCAAA-3′; *Eno1*-F, 5′-TAACGAGCAGGAAAGGAAGAC-3′; *Eno1*-R, 5′-AGGAGAGCCTTAAGGACAGA-3′; *Pgam1*-F, 5′-GTGGCAGAGACAGGAAATCT-3′; *Pgam1*-R, 5′-GACAGTCTCTCTGCGTAACC-3′; *Pgk1*-F, 5′-GACAGTCTCTCTGCGTAACC-3′; *Pgk1*-R, 5′-GTGAGACGTGCTACTTCCATTT-3′; *Pkm2*-F, 5′-GCAGCCAGCCTGTAAGGGCA-3′; and *Pkm2*-R, 5′-GCGAAGACAGGAAAACAGTGGGT-3′.

### α-Ketoglutarate (α-KG) Quantification

α-KG levels were quantified in monocytes isolated from the BM of four experimental groups of the IMQ-psoriasis mouse model. Measurements were performed using the α-KG Quantitation Kit (Sigma-Aldrich, Cat# MAK541) according to the manufacturer's instructions.

### KDM6A/B enzymatic activity

Nuclear proteins were extracted from isolated BM monocytes using the Nuclear Extraction Kit (Cat# ab113474, Abcam, Cambridge, UK), following the manufacturer's protocol. Protein concentrations were subsequently quantified using the Pierce™ BCA Protein Assay Kit (Cat# 23,225, Thermo Fisher Scientific, MA, USA). KDM6A/B enzymatic activity was measured using 1 µg of nuclear protein extract per sample with the KDM6A/B Activity Quantification Assay Kit (Cat# ab156911, Abcam), according to the manufacturer's instructions.

### Seahorse XFp metabolic flux analysis

On day 6, monocytes isolated from BM were seeded at a density of 5 × 10^5^ cells per well in Seahorse XF 8-well mini plates (Cat# 103,022–100, Agilent Technologies, CA, USA). Assay medium consisted of XF-RPMI supplemented with 1 mM pyruvate, 2 mM glutamine, and 10 mM glucose, adjusted to pH 7.4. Mitochondrial respiration and glycolytic function were assessed using the XF Cell Mito Stress Test Kit (Cat# 103,010–100, Agilent) and the XF Glycolysis Stress Test Kit (Cat# 103,020–100, Agilent), respectively. The final working concentrations of inhibitors used for the Cell Mito Stress Test were as follows: 1.5 µM oligomycin, 1 µM FCCP, and 0.5 µM rotenone/antimycin A. For the Glycolysis Stress Test, the following concentrations were used: 10 mM glucose, 1 µM oligomycin, and 50 mM 2-deoxyglucose (2-DG).

### Flow cytometry and intracellular staining

On day 6, tissues were collected from mice and processed into single-cell suspensions. Cells were incubated with an unlabeled anti-CD16/32 monoclonal antibody (clone 93, Cat# 14–0161-82, Invitrogen) for 10 min at 4 °C to block non-specific Fc receptor binding. After washing with FACS buffer (PBS containing 2% FBS and 2 mM EDTA), the cell pellets were resuspended in a fixable viability dye along with fluorochrome-conjugated surface antibodies and incubated for 30 min at 4 °C.

Monocytes were identified by surface staining with the following antibodies: CD11b (Cat# 101,212, clone M1/70, APC, BioLegend), CD45 (Cat# 103,147, clone 30-F11, BV711, BioLegend), Ly-6G (Cat# 127,618, clone 1A8, PE-Cy7, BioLegend), CD11c (Cat# 117,339, clone N418, BV650, BioLegend), MHCII (Cat# 107,628, clone M5/114.15.2, APC-Cy7, BioLegend), and Ly-6C (Cat# 128,012, clone HK1.4, PerCP-Cy5.5, BioLegend). To exclude T, NK 1.1, and B cells (Lin) from the analysis, anti-CD3 (Cat# 100,233, clone 17A2, BV510, BioLegend), and anti-NK 1.1 (Cat# 108,738, clone PK136, BV510, BioLegend) were included. For T cell analysis, cells were stained with antibodies against CD4 (Cat# 100,443, clone GK1.5, BV421, BioLegend), CD3 (Cat# 152,304, clone 500A2, FITC, BioLegend), CD8a (Cat# 100,714, clone 53–6.7, APC-Cy7, BioLegend), CD45 (Cat# 103,147, clone 30-F11, BV711, BioLegend), and TCRγδ (Cat# 118,124, clone GL3, PE-Cy7, BioLegend). Following surface staining, cells were fixed and permeabilized using the Foxp3/Transcription Factor Staining Buffer Set (Invitrogen, Cat# 5523), and intracellular staining was performed using fluorochrome-conjugated antibodies. For monocytes, intracellular markers included anti-TNF-α (Cat# 506,328, clone MP6-XT22, BV421, BioLegend), anti-Pro-IL1β (Cat# 12,711,482, clone NJTEN3, PE, Invitrogen), and anti-KDM6B (Cat# NBP1-06640AF488, AF488, Bio-Techne GmbH). T cells were intracellularly stained with antibodies against RORγT (Cat# 562,894, clone Q31-378, BV421, BioLegend), IL-17A (Cat# 506,920, clone TC11-18H10.1, PerCP/Cy5.5, BioLegend), Ki-67 (Cat# 151,215, clone 11F6, BV650, BioLegend), and Foxp3 (Cat# 12–5773-82, clone FJK-16 s, PE, eBioscience). All samples were acquired using a BD LSRII flow cytometer and analyzed using FlowJo v10.10.0 software (BD Biosciences).

### Keratinocyte viability assay

To assess cell viability, 2,000–3,000 murine keratinocytes were seeded per well in a white 96-well plate (Cat# 6,005,680, PerkinElmer), before the first measurement. Cells were exposed to 0, 1, 5, or 10 μM GSK-J4 for 6, 9, or 12 h, with 0 μM serving as the control. During the viability measurement, cells were incubated at 37 °C and 5% CO2. The CellTiter-Glo® Luminescent Cell Viability Assay (Cat#G7571, Promega) was performed according to the manufacturer’s instructions. Luminescence was detected using the Hidex Sense Microplate Reader. Relative luminescence units (RLU) retrieved from 0 μM treated cells were set to 100%, and the relative viability signal was assessed by calculating the fold change in RLUs compared to the 0 μM treated value.

### Single-cell RNA sequencing

C57BL/6 female mice were randomly assigned to three experimental groups: Sham, IMQ, and IMQ/GSK-J4, and treated as described above in two independent experiments. On day 6, spleen and skin tissues were harvested and further processed to generate single cell suspensions. Spleen was disrupted onto a filter mesh in a 3 cm Petri dish. Red blood cells were lysed using commercial RBC lysis buffer by incubation at room temperature for 3 min, followed by centrifugation for 2 min at 4 °C and 1000 rcf. Dorsal skin was excised, manually cut into small pieces using scissors and digested in DMEM (Cat#11,960,044, Gibco), 4 mg/ml collagenase type IV (Sigma-Aldrich, Cat#C5138), 20 μg/ml DNase I (Cat#89,836, Thermo Fisher Scientific) and 5 mg/ml bovine serum albumin (Carl Roth #90,604–29-8). Skin tissue was digested using gentleMACS C tubes (Miltenyi Biotec), and the ‘37C_Multi_H’ program was run on the gentleMACS dissociator. Murine samples were stained using the following surface anti-mouse antibodies: CD45-BUV737 (Cat#752,414, BD Biosciences), CD4-BV421 (Cat#566,644, BioLegend), CD8-PE-Cy7 (Cat#100,722, BioLegend), CD25-PE (Cat#102,008, BioLegend), CD11b-APC (Cat#101,212, BioLegend), CD11c-APC (Cat#117,310, BioLegend), CD19-APC (Cat#115,512, BioLegend), MHCII-APC (Cat #107,614, BioLegend), CD206-APC (Cat#141,708, BioLegend), NK1.1-APC (Cat#108,710, BioLegend) and Zombie NIR (Cat#423,106, BioLegend). Individual samples were stained with 6 TotalSeq™-C anti-mouse hashtag antibodies (BioLegend C1 #155,861 for Sham-treated mouse/spleen, C2 #155,863 for IMQ-treated mouse/spleen, C3 #155,865 for IMQ/GSK-J4-treated mouse/spleen, C4 #155,867 for Sham-treated mouse/skin, C5 #155,869 for IMQ-treated mouse/skin, C6 #155,871 for IMQ/GSK-J4-treated mouse/skin) at 4 °C for 20 min in 100 μL FACS buffer (StemCell Technologies Cat#07905). CD45⁺ immune cells were sorted for single-cell RNA sequencing. The following numbers of cells were sorted into 1.5 mL Eppendorf tubes containing 1 × PBS supplemented with 0.5% BSA and maintained at 4 °C: 8,000 CD45⁺ cells from spleen (Sham: TotalSeqC1; IMQ: TotalSeqC3; IMQ/GSK-J4: TotalSeqC5), and 4,000 CD45⁺ cells from skin (Sham: TotalSeqC2; IMQ: TotalSeqC4; IMQ/GSK-J4: TotalSeqC6). Following sorting, cells were centrifuged at 300 × g for 5 min at 4 °C, and the supernatant was carefully removed. The resulting cell pellets were resuspended in 37.5 µL of 0.5% BSA-PBS buffer. Cell suspensions were mixed with Master Mix from the Chromium Next GEM Single Cell 5′ v2 (Dual Index) kit with Feature Barcode Technology (Cat# 1,000,266, 10 × Genomics), and samples were loaded onto a Chromium Next GEM Chip K (Cat# 2,000,182, 10 × Genomics). Single-cell libraries were prepared according to the manufacturer's protocol (Chromium Next GEM Single Cell 5′ v2 with Feature Barcode Technology, CG000330 Rev F). The entire experiment was performed twice, and all libraries were sequenced using an Illumina NextSeq™ 550 system with a 150-cycle high-output cartridge.

### Bioinformatics analysis

Initial data processing, quality control, and integration followed the previously established protocol described by Nedwed et al. [20]. Raw sequencing reads were demultiplexed and aligned using Cell Ranger v7.1.0 (10 × Genomics) [21], employing a transcriptome index generated from the Mus musculus genome build GRCm38. Gene annotation was based on ENSEMBL release 102 for Mus musculus. Quality control (QC) was performed independently on each dataset to remove poor-quality cells using scater v1.32.0 [22]. Mitochondrial gene content served as a proxy for identifying damaged cells, using a threshold of three median absolute deviations above the median, in accordance with recommendations from the OSCA guidelines [23]. Doublet detection was performed with scDblFinder v1.18.0 [24]. After QC, normalization of cell-specific biases was conducted using the deconvolution-based method implemented in scran [25]. Counts were divided by size factors, and normalized values were log-transformed after adding a pseudocount of one. Integration of datasets from different biological samples was achieved using the Mutual Nearest Neighbors (MNN) method implemented in batchelor v1.20.0 [25]. Subsequently, highly variable genes (HVGs) were identified by decomposing per-gene variability into technical and biological components based on the mean–variance trend. Dimensionality reduction was performed using Principal Component Analysis (PCA). PCA results were then used as input for t-distributed stochastic neighbor embedding (t-SNE) for visualization [26]. Clustering analysis utilized the 9,982 most highly variable genes to build a shared nearest neighbor graph (SNNG) [27]. Clusters were determined using the Louvain community detection algorithm implemented via igraph (https://doi.org/10.5281/zenodo.7682609).

Initial automated cell-type annotation was performed using SingleR v2.6.0 [28] with the ImmGenData reference from celldex v1.14.0 [28]. These annotations were manually refined using established cell-type marker genes from the literature. Visualization and manual refinement of cell clusters were carried out using iSEE v2.16.0 [29] and iSEEfier v1.2.0 (https://bioconductor.org/packages/iSEEfier). Most visualizations for single-cell data were generated using iSEE. Pseudobulk differential gene expression analysis was conducted using muscat v1.20.0 [30] with the default modeling approach. Multiple testing corrections were applied using the Benjamini-Hochberg (BH) method, and genes with adjusted p-values < 0.05 were considered DEGs. Functional enrichment analysis of DEGs was performed with mosdef v1.2.0 (https://bioconductor.org/packages/mosdef), implementing the topGO package [31]. Enrichment analysis used the "elim" algorithm within the Biological Process ontology, employing DEGs as input against a background set comprising all detected genes. Finally, differential abundance analysis comparing cell type proportions across treatment conditions was performed using speckle v1.6.0 [32]. Statistical significance was defined at a False Discovery Rate (FDR) < 0.05.

### Statistical analysis

The sample size, number of replicates, and detailed statistical information for each experiment are provided in the figure legends. All data are presented as mean ± SEM, as specified in the respective figure legends unless otherwise stated. The Shapiro–Wilk test was used to assess normality, and the Brown–Forsythe or F test was used to assess homogeneity of variance, as appropriate. Statistical analysis was performed using one-way ANOVA followed by post hoc analysis (Tukey’s multiple comparison test) for pairwise comparisons among more than two groups. Longitudinal scores were analyzed using two-way repeated-measures ANOVA with Geisser-Greenhouse correction. Statistical analyses were performed in GraphPad Prism (v6) using the tests described above.

## Results

### Monocyte depletion reduces IMQ-induced psoriasis severity

**Fig. 1:**
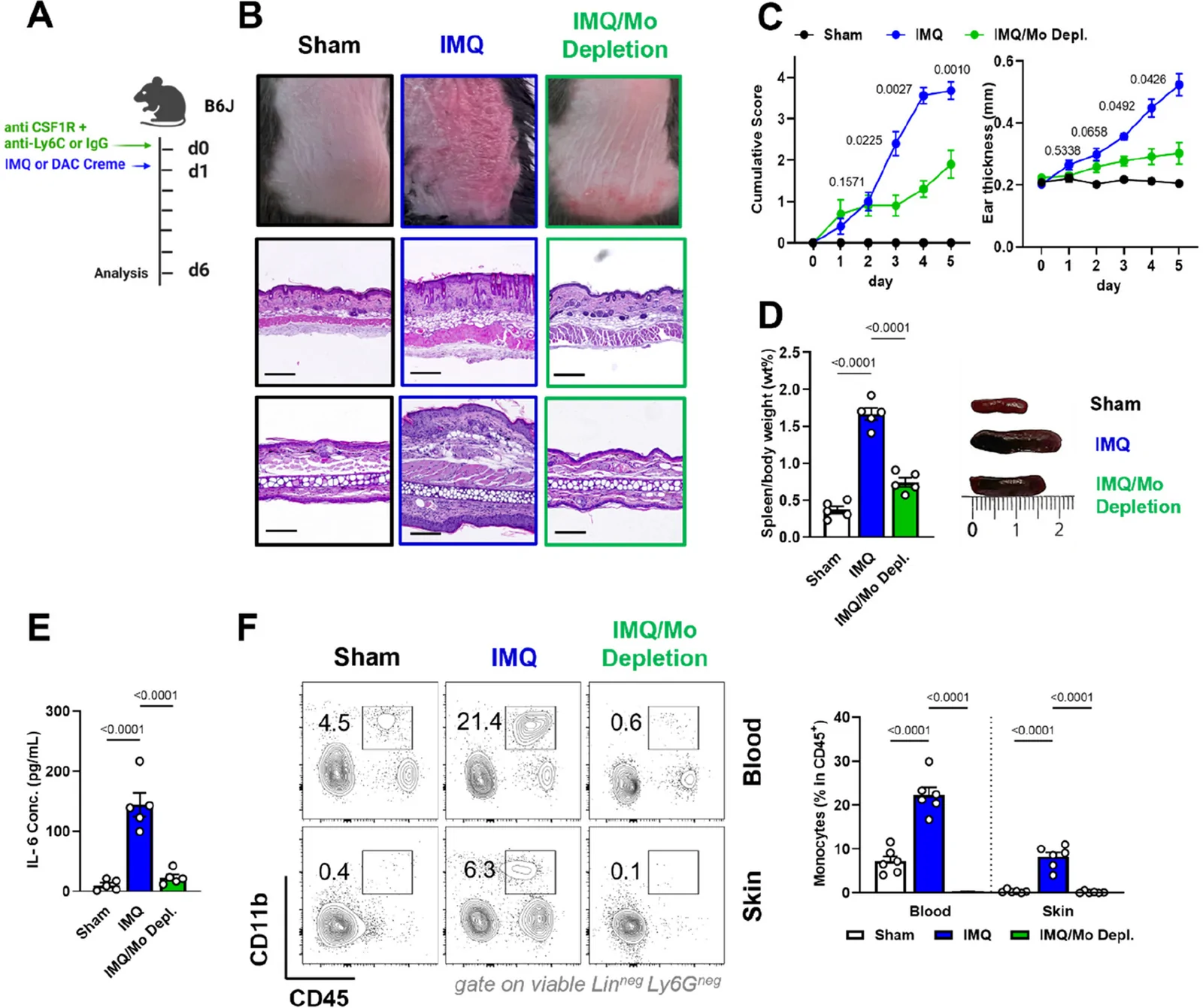
Monocyte depletion attenuates local and systemic inflammation in the IMQ-induced psoriasis model. **A** Experimental schematic for in vivo monocyte depletion via anti-CSF1R and anti-Ly6C antibodies followed by IMQ application. **B** Representative images and H&E-stained skin sections from control, IMQ-treated and IMQ/monocyte-depleted mice. Scale bars: 100–200 µm. **C** Clinical scores for erythema and scaling (left) as well as quantification of ear thickness (right) over six days of IMQ treatment. **D** Spleen-to-body-weight ratio in the indicated treatment groups. **E** Plasma IL-6 concentrations measured by ELISA. **F** Representative flow cytometry plots showing frequencies of CD11b⁺Ly6G⁻ monocytes in blood and skin. Data are representative of 2 independent experiments (*n* = 6 mice per group). Longitudinal data were analyzed using two-way repeated-measures ANOVA with Geisser-Greenhouse correction. Other comparisons were analyzed by one-way ANOVA followed by the Tukey’s multiple comparison test. Data are presented as mean ± SEM

To better understand the role of monocytes in the IMQ-induced psoriasis model, we first examined the effect of monocyte depletion on disease onset and severity. Mice were treated daily with an intraperitoneal injection of depleting antibodies directed against CSF1R (CD115) and Ly6C (100 µg each per mouse) or the corresponding isotype IgG controls, starting one day before topical challenge (day 0) and continuing throughout the experiment **(**Fig. 1A**).** Antibody treatment markedly reduced circulating Ly6C⁺ monocytes, while exerting only a limited effect on circulating Ly6G⁺ neutrophils. Because CSF1R and Ly6C are also expressed by other myeloid populations, including subsets of macrophages and neutrophils, potential off-target effects of the depletion strategy cannot be excluded (Fig. S1A). From day 1 to day 5, 5% IMQ cream (Aldara) or vehicle control DAC cream was applied once daily to both ears (10 mg per ear) and to the shaved dorsal skin (50 mg) **(**Fig. 1A**)**. Monocyte depletion markedly ameliorated IMQ-induced skin inflammation by day 6, as evidenced by reduced erythema and scaling **(**Fig. 1B-C**)**. Clinical scoring throughout the treatment period further demonstrated reductions in erythema, scaling, and skin thickening following monocyte depletion **(**Fig. 1B-C**).** Histological analysis revealed a significant reduction in epidermal thickness and inflammatory cell infiltration in monocyte-depleted, IMQ-treated mice compared with IMQ-treated control mice **(**Fig. 1B**).** Monocyte depletion also significantly decreased spleen weight **(**Fig. 1D**)**, consistent with reduced systemic immune activation. Furthermore, plasma cytokine analysis by ELISA revealed significantly lower IL-6 concentrations in monocyte-depleted mice compared with IMQ-treated controls **(**Fig. 1E**).** Flow cytometric analysis demonstrated reduced frequencies of CD11b⁺Ly6G⁻ monocytes in both the blood and skin **(**Fig. 1F and S1D**).** Together, these findings indicate that depletion of Ly6C⁺ monocytes is associated with attenuation of both local IMQ-induced skin inflammation and systemic inflammatory responses.

### KDM6B expression is elevated in monocytes in the IMQ-induced psoriasis model

**Fig. 2:**
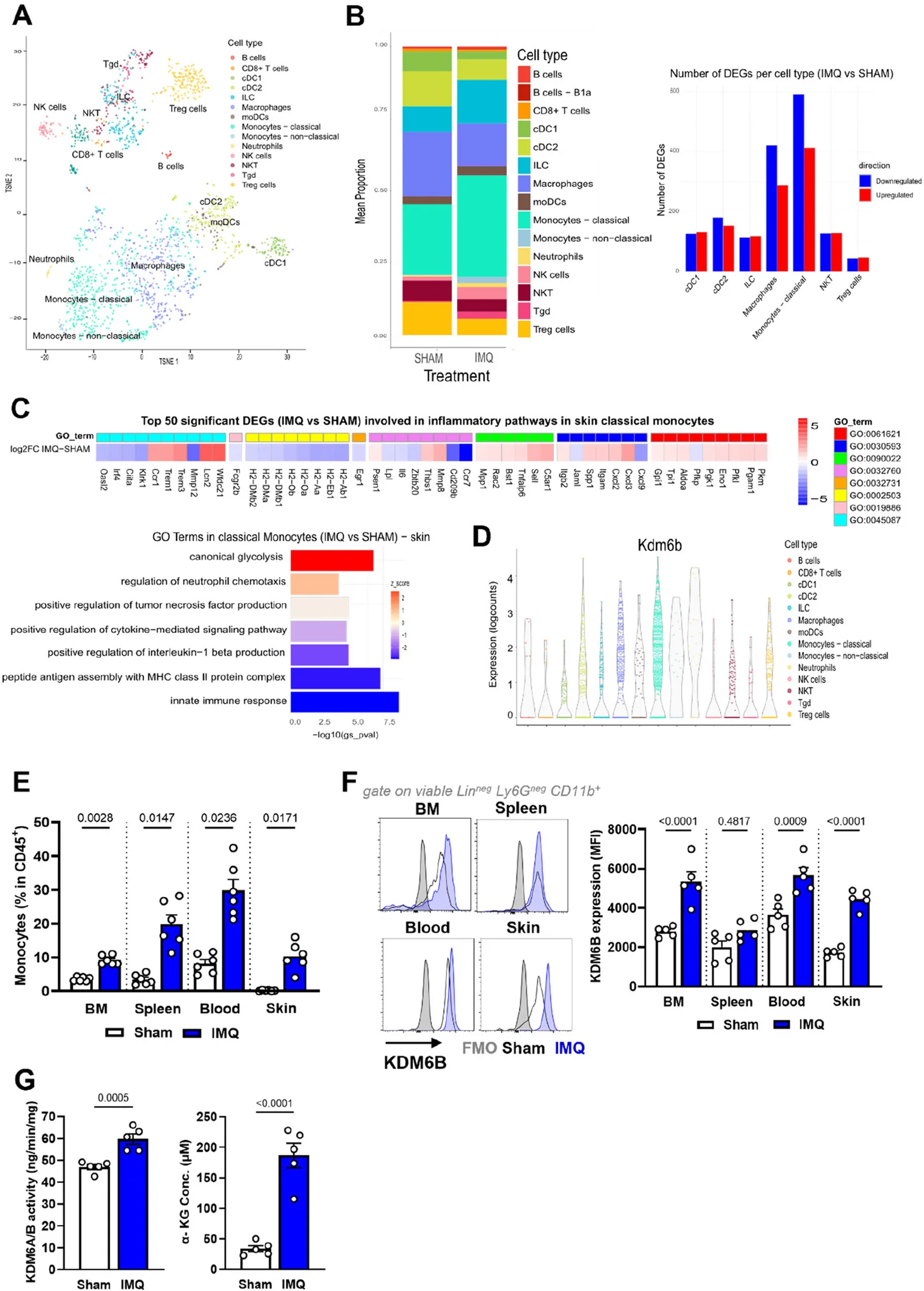
KDM6B expression and α-KG levels are increased in monocytes during IMQ-induced psoriasis-like inflammation. **A** t-SNE plot of CD45⁺ immune cells isolated from the skin of IMQ-treated and sham mice. **B** Relative abundance of immune cell subsets in sham-treated versus IMQ-treated skin (left) and number of differentially expressed genes (DEGs) across immune populations (right). **C** Heatmap of DEGs and GO enrichment analysis of upregulated and downregulated genes in classical monocytes from IMQ versus sham skin. **D** *Kdm6b* expression across the indicated immune-cell populations in sham- and IMQ-treated skin. **E** Representative flow cytometry plots showing frequencies of CD11b⁺Ly6G⁻ monocytes in BM, spleen, blood, and skin. **F** Representative flow cytometry histograms and quantification of intracellular KDM6B protein expression in BM, spleen, blood, and skin from sham- and IMQ-treated mice. Data are presented as normalized modal mean fluorescence intensity (MFI). **G** KDM6A/B demethylase activity and intracellular α-KG levels in BM monocytes from sham- and IMQ-treated mice. Data are representative of three independent experiments (*n* = 5–6 mice per group). For E–G, statistical analysis was performed using an unpaired t-test. Data are presented as the mean ± SEM

To dissect the cellular landscape and functional states of immune cells in the IMQ-induced psoriasis model, we performed single-cell RNA sequencing (scRNA-seq) on CD45⁺ cells isolated from the skin and spleen of IMQ-treated and sham-treated mice. Individual replicates, treatment regimens, and tissue types were distinguished using hashtag oligo-based barcoding of samples. Dimensionality reduction using t-distributed stochastic neighbor embedding (t-SNE) revealed transcriptionally distinct clusters corresponding to major immune cell types, including classical (*Itgam*, *Ly6c2*) and non-classical monocytes (*Itgam*, *Spn*, with lower *Ly6c2* expression), neutrophils (*Ly6g*), macrophages (*Adgre1*, *Fcgr1*), dendritic cells (for whole DCs: *Zbtb46 and Itgax*, cDC1: *Xcr1*; cDC2: *Sirpa,* moDCs: *Cd209a*, *H2-Ab1*), CD4⁺ and CD8⁺ T cells (*Cd3e, Cd4, Cd8a*), Tregs (*Foxp3*), Th17 cells (*Cd4, Il17a, Rorc*), NK cells (*Ncr1*), ILCs (*Gata3, Rorc*), and B cells (*Cd19*, *Ms4a1*) **(**Fig. 2A and S1B**).**

Comparison of immune-cell proportions between IMQ-treated and sham-treated skin showed an increased proportion of classical monocytes following IMQ treatment **(**Fig. 2B and S1C**)**. Differential expression analysis further revealed pronounced transcriptional alterations across myeloid populations, particularly classical monocytes and macrophages **(**Fig. 2B**)**. Differential gene expression analysis of classical monocytes from IMQ-treated versus sham-treated skin identified changes in genes associated with inflammatory and metabolic processes **(**Fig. 2C**).** Gene Ontology (GO) enrichment analysis of upregulated genes identified enrichment of terms associated with canonical glycolysis (GO:0061621), neutrophil chemotaxis (GO:0030593 and GO:0090022), TNF production (GO:0032760), and IL-1β signaling (GO:0032731). Conversely, downregulated genes were enriched for GO terms related to MHC class II-dependent antigen processing and presentation (GO:0002503 and GO:0019886) and innate immune responses (GO:0045087) **(**Fig. 2C**).** Together, these transcriptional changes indicate that classical monocytes acquire a distinct inflammatory and metabolic transcriptional state during IMQ-induced skin inflammation.

Based on our previous observations of increased KDM6B expression in peripheral monocytes from patients with psoriasis [16, 17], we next examined KDM6B expression in the IMQ-induced psoriasis model. Consistent with these human data, scRNA-seq analysis showed increased *Kdm6b* expression in classical monocytes and other myeloid populations in IMQ-treated skin compared with sham-treated skin **(**Fig. 2D**).** We next validated the increase in monocyte abundance and KDM6B expression at the protein level across multiple tissues [5, 8, 10]. Monocytes were analyzed in BM, spleen, blood, and skin of mice treated with IMQ for six days, as well as sham controls, for subsequent analyses. Flow cytometry revealed an increased frequency of CD11b⁺Ly6G⁻ monocytes among CD45⁺ cells in the indicated tissues **(**Fig. 2E and S1D**).** KDM6B protein expression was also increased in CD11b⁺ monocytes (gated on viable CD45⁺Lin⁻ [CD3⁻CD19⁻NK1.1⁻] Ly6G⁻ cells) in the analyzed tissues following IMQ treatment **(**Fig. 2F**)**. Finally, KDM6A/B demethylase activity and intracellular α-ketoglutarate (α-KG) levels were increased in BM monocytes from IMQ-treated mice compared with sham controls **(**Fig. 2G**).** Because α-KG is a required co-substrate for JmjC-domain-containing histone demethylases, including KDM6B [33], these findings are consistent with increased availability of a metabolite that supports KDM6 catalytic activity. These results demonstrate that increased KDM6B expression during IMQ-induced inflammation is accompanied by increased KDM6A/B enzymatic activity and increased availability of a metabolite required for KDM6B catalytic activity.

### GSK-J4 treatment attenuates IMQ-induced psoriasis-like inflammation after disease onset

**Fig. 3:**
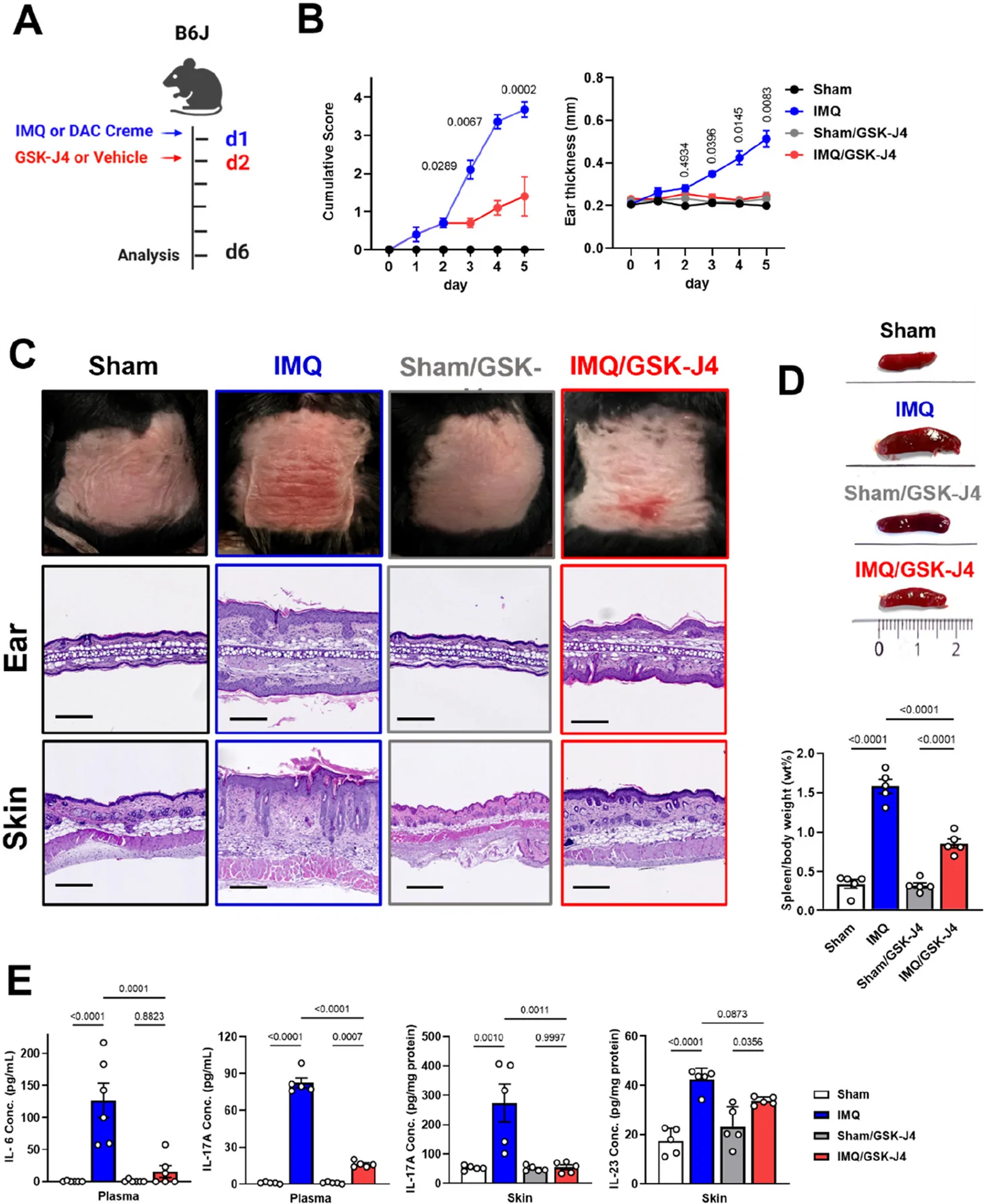
GSK-J4 treatment attenuates IMQ-induced psoriasis-like inflammation after disease onset. **A** Treatment scheme of the IMQ-induced psoriasis-like inflammation model. Intraperitoneal vehicle or GSK-J4 injection started on day 2 of the IMQ course (post-onset). **B** Longitudinal assessment of erythema and scaling scores and ear thickness in the indicated treatment groups (*n* = 12). **C** Representative macroscopic images of sham-treated, IMQ-treated, and IMQ/GSK-J4-treated mice. H&E staining of ears and dorsal skin on day 6. Scale bars: 100 µm. **D** Spleen-to-body-weight ratio in the indicated treatment groups. **E** Plasma IL-6 and IL-17A concentrations and skin IL-17A and IL-23 concentrations measured by ELISA (*n* = 6–8 mice per group). Data are representative of 4 independent experiments. Data are presented as mean ± SEM; statistical significance was assessed using one-way ANOVA with the Tukey’s multiple comparison test. For B, statistical analysis was performed using two-way repeated-measures ANOVA with Geisser-Greenhouse correction

GSK-J4 is a cell-permeable prodrug of GSK-J1, an inhibitor of H3K27 demethylases, and has been investigated in inflammatory diseases such as SLE and abdominal aortic aneurysm, showing promising efficacy both in vitro and in vivo [14, 18]. However, it remains unclear whether KDM6B plays a critical role in psoriasis-induced inflammation in the IMQ-induced psoriasis model and whether KDM6B inhibition could effectively alleviate established skin inflammation. To address this question, mice were treated topically with IMQ-containing cream for five consecutive days. Beginning on day 2, after the onset of clinical signs, mice additionally received intraperitoneal injections of either GSK-J4 (10 mg/kg) or vehicle control (DMSO) **(**Fig. 3A**)**.

Systemic administration of GSK-J4 significantly attenuated the progression of IMQ-induced skin inflammation, as demonstrated by reduced ear thickness and lower clinical scores for erythema and scaling **(**Fig. 3B-C, and Fig. S2A**).** Macroscopic examination and histological analysis of dorsal skin and ears at day 6 further demonstrated reduced skin inflammation in GSK-J4-treated mice compared with vehicle-treated IMQ controls **(**Fig. 3C**)**. Histologically, GSK-J4 treatment reduced IMQ-induced epidermal thickening and inflammatory cell infiltration **(**Fig. 3C**)**. GSK-J4 treatment also reduced the IMQ-induced increase in spleen weight (spleen-to-body-weight ratio) **(**Fig. 3D**)**. Furthermore, plasma concentrations of IL-6 and IL-17A and skin concentrations of IL-17A and IL-23 were reduced following GSK-J4 treatment **(**Fig. 3E**)**. Together, these findings demonstrate that systemic GSK-J4 treatment initiated after the onset of clinical signs attenuates both local skin pathology and systemic inflammatory manifestations in the IMQ-induced psoriasis model.

### GSK-J4 treatment reduces myeloid accumulation and inflammatory immune responses in IMQ-induced psoriasis-like inflammation

**Fig. 4:**
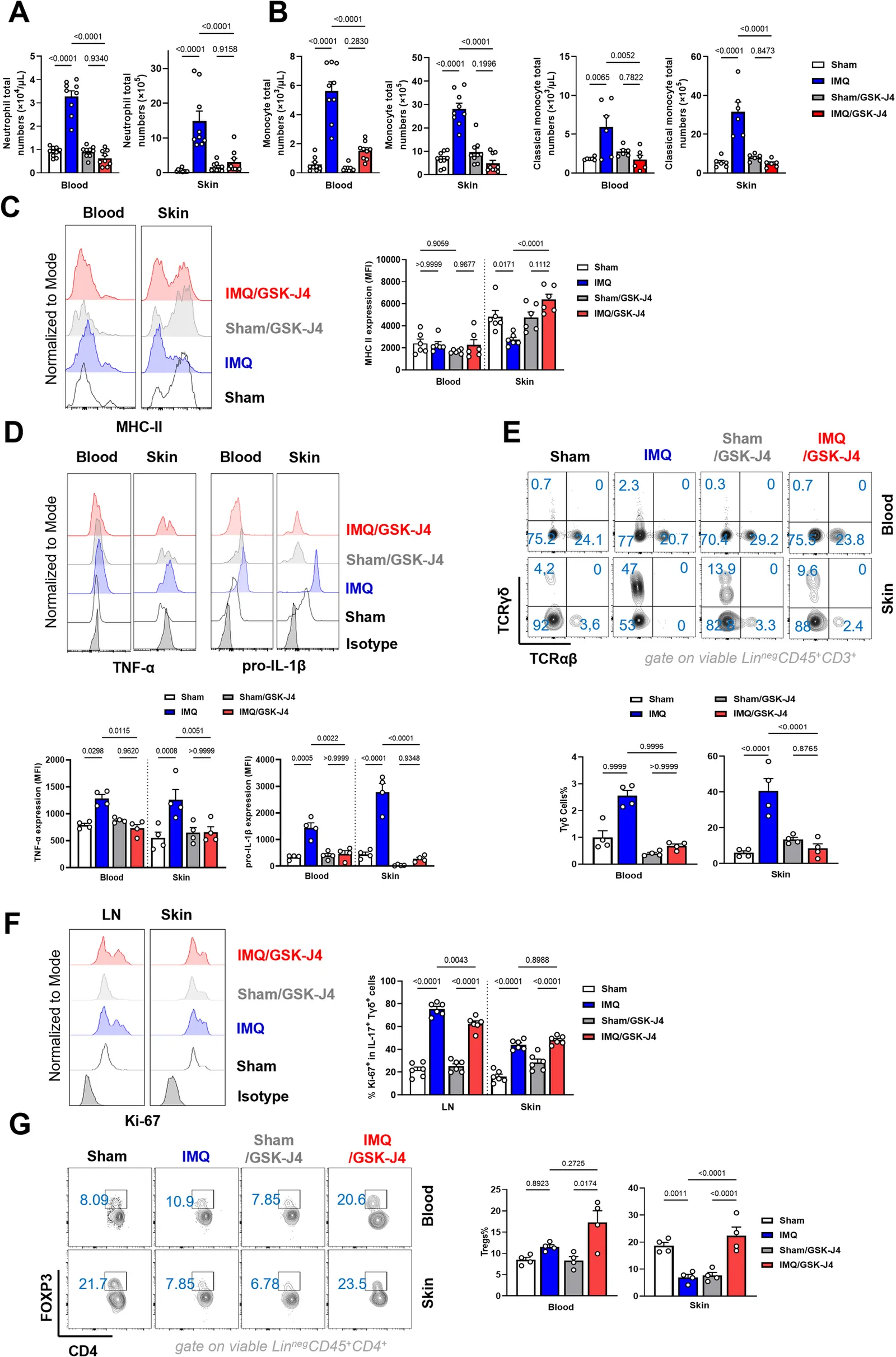
GSK-J4 treatment alters myeloid and lymphoid immune responses during IMQ-induced psoriasis-like inflammation. **A** Absolute numbers of neutrophils (viable CD45⁺Lin⁻ CD11b⁺ Ly6G⁺ cells) in the blood and skin of sham-, IMQ-, sham/GSK-J4-, and IMQ/GSK-J4-treated mice, quantified by flow cytometry (*n* = 6–8). **B** Absolute numbers of monocytes (viable CD45⁺Lin⁻ CD11b⁺ Ly6G⁻ cells) and classical monocytes (viable CD45⁺Lin⁻ CD11b⁺ Ly6G⁻Ly6C⁺ cells) in the same groups, quantified by flow cytometry (*n* = 6–8). **C** Representative flow cytometry histograms and quantification of MHC-II expression in monocytes from blood and skin. Data are presented as normalized modal MFI. **D** Representative flow cytometry histograms and quantification of intracellular TNF-α and pro-IL-1β expression in monocytes from blood and skin. Data are presented as normalized modal MFI. **E** Frequencies of γδ T cells (viable CD45⁺CD3⁺TCRαβ⁻TCRγδ⁺ cells) in blood and skin and (**F**) Ki-67 expression of IL-17A-producing γδ T cells in inguinal draining lymph nodes (LN) and skin were assessed by flow cytometry. **G** Flow cytometric quantification of CD4⁺FOXP3⁺ Tregs (viable CD45⁺CD3⁺) in blood and skin. Data are representative of four independent experiments (*n* = 4–6 mice per group) and are presented as mean ± SEM. Statistical significance was determined using one-way ANOVA with the Tukey’s multiple comparison test

Myeloid cell infiltration into the skin and peripheral tissues is a hallmark of IMQ-psoriasis and is closely associated with disease progression [5, 6]. To examine immune-cell changes associated with GSK-J4 treatment, we examined myeloid and lymphoid immune-cell populations in the blood, skin, and draining lymph nodes of IMQ-treated mice. Flow cytometry analysis revealed that IMQ-induced psoriasis increased the abundance of neutrophils (viable CD45⁺Lin⁻ [CD3⁻CD19⁻NK1.1⁻] CD11b⁺Ly6G⁺) **(**Fig. 4A**)**, total monocytes (viable CD45⁺Lin⁻ [CD3⁻CD19⁻NK1.1⁻] CD11b⁺Ly6G⁻), and classical monocytes (viable CD45⁺Lin⁻Ly6G⁻CD11b⁺Ly6C⁺) **(**Fig. 4B**)** in both blood and skin, which were significantly reduced following GSK-J4 treatment. Interestingly, GSK-J4 also increased MHC-II expression on monocytes **(**Fig. 4C**)** and reduced CD11c⁺MHC-II⁺ cells in IMQ-treated mice **(**Fig. S2B**).** Together, these data show that GSK-J4 treatment alters the accumulation and activation phenotype of multiple myeloid-cell populations during IMQ-induced inflammation. Intracellular cytokine staining further showed reduced TNF-α and pro-IL-1β expression in monocytes from the blood and skin of IMQ/GSK-J4-treated mice compared with IMQ-treated controls **(**Fig. 4D**)**. Thus, in addition to reducing monocyte accumulation, GSK-J4 treatment was associated with decreased expression of pro-inflammatory cytokines by the remaining monocyte population.

Since monocytes have been shown to shape downstream adaptive immune responses, including γδ T cells and Tregs in the IMQ-psoriasis mouse model [5, 34], we examined these populations.

Consistent with the predominant role of γδ T cells as the major IL-17-producing population in the IMQ model, the frequency of γδ T cells (viable CD45⁺CD3⁺TCRαβ⁻TCRγδ⁺ cells) in the skin was reduced following GSK-J4 treatment **(**Fig. 4E**)**. Analysis of Ki-67 expression in IL-17A-producing γδ T cells revealed no significant difference in the skin, whereas Ki-67 expression in IL-17A-producing γδ T cells from inguinal draining lymph nodes was significantly reduced in IMQ/GSK-J4-treated mice compared with IMQ-treated controls **(**Fig. 4F**)**. These findings indicate tissue-specific effects of GSK-J4 on the proliferative state of IL-17A-producing γδ T cells. In contrast, GSK-J4 treatment increased the frequency of CD4⁺FOXP3⁺ Tregs (viable CD45⁺CD3⁺) in the blood and skin of IMQ-treated mice **(**Fig. 4G**)**, suggesting a shift toward an immunoregulatory milieu. This observation supports the hypothesis that GSK-J4 treatment was associated with increased Treg frequency, consistent with a shift in the immune-cell composition toward a more regulatory phenotype [35–37]. However, the possibility that GSK-J4 directly influences γδ T cells and Tregs cannot be excluded.

Because systemic GSK-J4 treatment could also directly affect non-hematopoietic cells, we assessed keratinocyte viability following GSK-J4 exposure in vitro. GSK-J4 reduced keratinocyte viability in a concentration-dependent manner, with a significant reduction observed at concentrations of ≥ 5 μM **(**Fig. S2C**)**. These findings indicate that GSK-J4 can directly affect keratinocytes at higher concentrations and therefore support caution in attributing its in vivo effects exclusively to immune cells. Collectively, these results demonstrate that GSK-J4 treatment broadly remodels inflammatory immune responses during IMQ-induced psoriasis-like inflammation, reducing neutrophil and monocyte accumulation, decreasing pro-inflammatory cytokine expression in monocytes, reducing γδ T-cell responses, and increasing Treg frequencies.

### GSK-J4 attenuates IL-36α/IL-17A-induced psoriasis-like inflammation

**Fig. 5:**
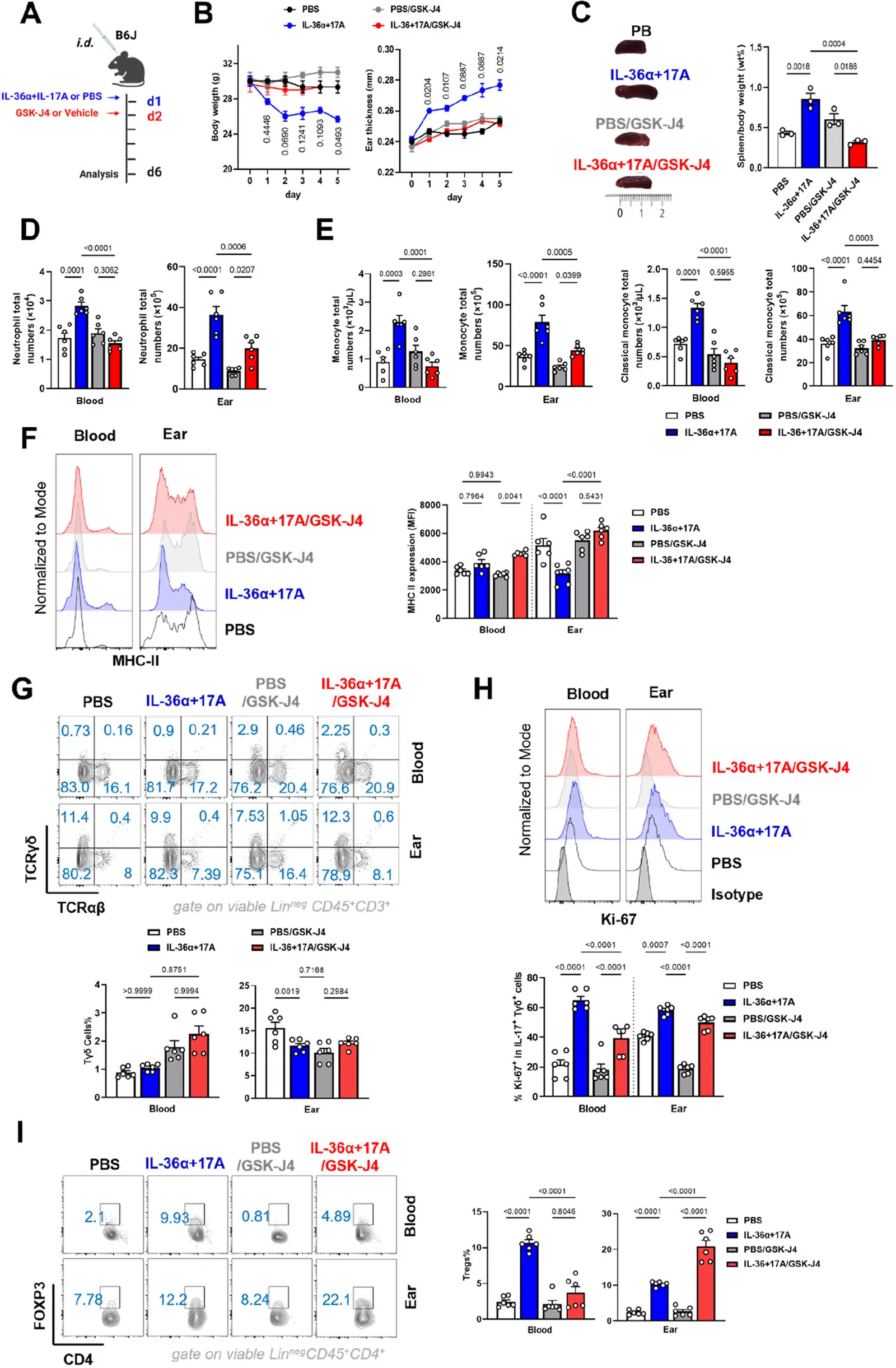
GSK-J4 attenuates IL-36α/IL-17A-induced psoriasis-like inflammation and remodels inflammatory immune-cell responses. **A** Treatment scheme of the IL-36α/IL-17A-induced psoriasis-like inflammation model and GSK-J4 treatment. **B** Longitudinal assessment of body weight and ear thickness in PBS-, IL-36α + IL-17A-, PBS/GSK-J4-, and IL-36α + IL-17A/GSK-J4-treated mice. **C** Representative spleen images and quantification of spleen weight or spleen-to-body-weight ratio. **D** Absolute numbers of neutrophils (viable CD45⁺Lin⁻ CD11b⁺ Ly6G⁺ cells) in the blood and ears of PBS-, IL-36α + IL-17A-, PBS/GSK-J4-, and IL-36α + IL-17A/GSK-J4-treated mice, quantified by flow cytometry. **E** Absolute numbers of monocytes (viable CD45⁺Lin⁻ CD11b⁺ Ly6G⁻ cells) and classical monocytes (viable CD45⁺Lin⁻ CD11b⁺ Ly6G⁻Ly6C⁺ cells) in the same groups, quantified by flow cytometry. **F** Representative flow cytometry histograms and quantification of MHC-II expression in monocytes from blood and ears. Data are presented as normalized modal MFI. **G** Representative flow cytometry plots and quantification of γδ T cells (viable CD45⁺CD3⁺TCRαβ⁻TCRγδ⁺ cells) in blood and ears. **H** Representative histograms and quantification of Ki-67 expression in IL-17A-producing γδ T cells in blood and ears. **I** Representative flow cytometry plots and quantification of CD4⁺FOXP3⁺ Tregs (viable CD45⁺CD3⁺) in blood and ears. Data are representative of two independent experiments (*n* = 6 mice per group) and are presented as mean ± SEM. Statistical significance was determined using one-way ANOVA with Tukey’s multiple comparison test. For B, statistical analysis was performed using two-way repeated-measures ANOVA with Geisser-Greenhouse correction

To determine whether the anti-inflammatory effects of GSK-J4 were restricted to the IMQ model or were also observed in a cytokine-driven model of psoriasis-like inflammation, we employed a model induced by repeated intradermal administration of IL-36α and IL-17A into the ears for 5 days. Mice received IL-36α and IL-17A or PBS, followed by treatment with GSK-J4 or vehicle beginning on day 2 **(**Fig. 5A**)**. Administration of IL-36α and IL-17A induced a progressive inflammatory phenotype, whereas GSK-J4 treatment attenuated disease progression, as reflected by attenuated body-weight loss and reduced ear swelling compared with cytokine-treated controls **(**Fig. 5B**)**. GSK-J4 treatment also reduced the cytokine-induced increase in spleen weight, consistent with attenuation of systemic inflammatory responses **(**Fig. 5C**)**.

We next investigated whether GSK-J4 altered the myeloid response induced by IL-36α and IL-17A. Flow cytometric analysis demonstrated that cytokine administration markedly increased the absolute numbers of neutrophils, total monocytes, and classical monocytes in blood and ears. These increases were attenuated following GSK-J4 treatment **(**Fig. 5D-E**)**. In parallel, MHC-II expression increased in monocytes from GSK-J4-treated mice compared with mice receiving IL-36α/IL-17A alone **(**Fig. 5F**)**. These findings indicate that GSK-J4 limits the accumulation of inflammatory myeloid populations in the cytokine-driven model.

We next examined the γδ T-cell compartment. GSK-J4 treatment increased the frequency of γδ T cells in blood but not in ears **(**Fig. 5G**)**. Representative flow cytometry plots and quantitative analysis further showed decreased proliferative activity of IL-17A-producing γδ T cells, as assessed by Ki-67 expression only in blood **(**Fig. 5H**)**. These findings suggest that GSK-J4 modulates γδ T-cell responses in addition to its effects on the myeloid compartment. We also assessed Tregs to determine whether GSK-J4 altered the balance between inflammatory and immunoregulatory lymphocyte populations. IL-36α/IL-17A treatment altered the abundance of CD4⁺FOXP3⁺ Tregs, whereas GSK-J4 treatment increased Treg frequency in ears **(**Fig. 5I**)**. Thus, similar to the IMQ model, GSK-J4 treatment was associated with a broader remodeling of the inflammatory immune-cell compartment, characterized by reduced myeloid-cell accumulation and increased representation of Treg cells. Collectively, these findings demonstrate that the anti-inflammatory effects of GSK-J4 are reproduced in an IL-36α/IL-17A-driven model of psoriasis-like inflammation, demonstrating anti-inflammatory activity of GSK-J4 across two mechanistically distinct models.

### GSK-J4 remodels classical monocyte and Treg transcriptional programs in IMQ-induced psoriasis-like inflammation

**Fig. 6:**
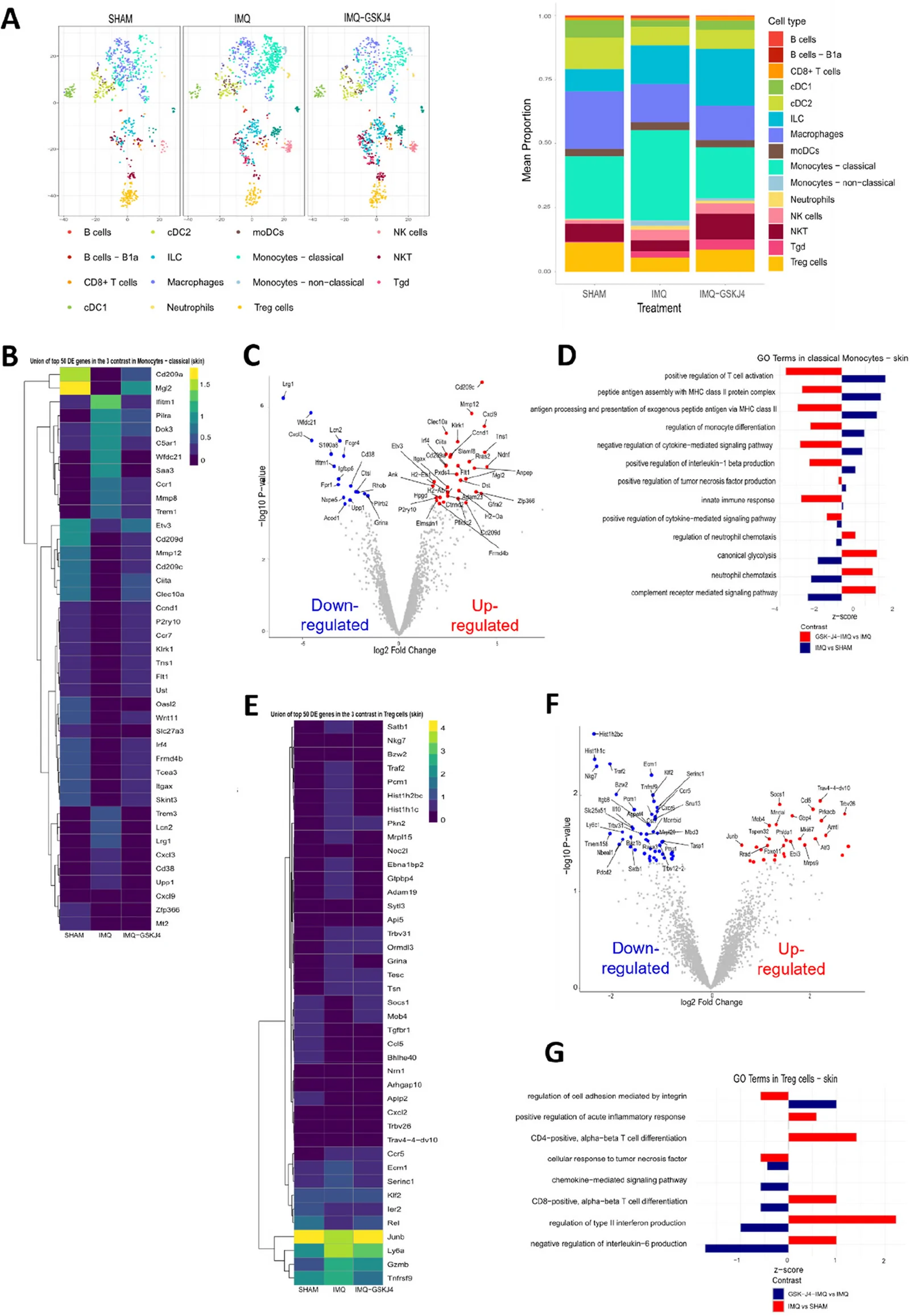
Single-cell transcriptional profiling reveals reduced inflammatory pathway expression in classical monocytes following GSK-J4 treatment in the IMQ-induced psoriasis-like mouse model. **A** t-SNE plots showing major immune cell populations identified from CD45⁺ cells isolated from the skin of sham-, IMQ-, and IMQ/GSK-J4-treated mice. Each dot represents a single cell and is colored according to annotated cell type; separate panels show the treatment conditions. Stacked bar plot showing the relative abundance of immune cell subsets. **B** Heatmap showing pseudobulk expression of the top 50 DEGs in classical monocytes across sham, IMQ, and IMQ/GSK-J4 groups; filtered heatmap of adjusted-P DEGs (*n* = 53) shown as indicated. **C** Volcano plot of DEGs in classical monocytes comparing IMQ/GSK-J4 versus IMQ groups. **D** GO enrichment analysis of DEGs in classical monocytes (IMQ vs IMQ/GSK-J4 groups). Red bars indicate pathways upregulated in IMQ, and blue bars indicate pathways enriched in IMQ/GSK-J4. **E** Heatmap showing the top 50 DEGs in Tregs from the same conditions. Gene expression levels are normalized and color-coded according to relative expression intensity. **F** Volcano plot of DEGs in Tregs comparing IMQ/GSK-J4 versus IMQ groups. Volcano plots display log₂ fold change versus -log₁₀ (adjusted P). **G** GO enrichment analysis of DEGs in Tregs from IMQ vs. IMQ/GSK-J4 conditions

Recent studies have demonstrated that GSK-J4 significantly reduces the expression of proinflammatory cytokine genes, including *Il6*, *Il1b*, *Tnf*, and *Il12* in monocytes and macrophages [14, 18, 37–39]. To comprehensively understand the immune landscape and the broader impact of systemic GSK-J4 on immune cells in IMQ-treated mice, we performed single-cell RNA sequencing (scRNA-seq) on isolated CD45⁺ immune cells from the skin and spleen of sham-treated, IMQ-treated, and IMQ/GSK-J4-treated mice. Dimensionality reduction using t-SNE revealed major immune cell subsets, including classical and non-classical monocytes, neutrophils, macrophages, DCs, CD4⁺ and CD8⁺ T cells, Tregs, NK cells, and B cells in the skin **(**Fig. 6A**)** and spleen (Fig. S3A). Prominently, treatment with GSK-J4 led to a significant reduction in classical monocyte abundance in the skin, partially restoring the cellular composition toward the homeostatic distribution observed in sham-treated skin **(**Fig. 6A**)**.

To assess the impact of KDM6B inhibition on IMQ-induced inflammatory reprogramming in classical monocytes, we performed pseudobulk differential gene-expression analysis across the experimental groups. The heatmap representing the top 50 DEGs (differentially expressed genes) in skin monocytes, selected from the union of significant DEGs (adj. p value < 0.05) across the three contrasts (IMQ/GSK-J4-treated vs sham-treated; IMQ-treated vs sham-treated; IMQ/GSK-J4-treated vs IMQ-treated) demonstrates that KDM6B inhibition markedly alters the transcriptional profile of classical monocytes **(**Fig. 6B**)**. IMQ alone induced a robust proinflammatory signature, characterized by upregulation of key mediators such as *Ifitm1, C5ar1, Saa3, Mmp8, Trem1/3, Lcn2, Lrg1, Cxcl3,* and *Cd38*. In contrast, GSK-J4 significantly suppressed this hyperinflammatory program, shifting toward a less inflammatory transcriptional state **(**Fig. 6B**)**.

The volcano plot **(**Fig. 6C**)**, comparing classical monocytes from IMQ/GSK-J4 versus IMQ treatment, highlights this transcriptional reprogramming. A total of 563 genes were significantly differentially expressed (adj. *P* < 0.05). Among these, several proinflammatory genes were significantly downregulated (e.g., *Ifitm1, S100a8, Cxcl3, Fpr1, Lrg1, Cd38, Lcn2,* and *Acod1*), while genes associated with antigen presentation (e.g., *H2-Aa, H2-Ab1, Slamf8, Ciita,* and *Mgl2*) were upregulated, indicating increased expression of antigen-presentation-associated genes **(**Fig. 6C**)**. Importantly, the heatmap of adjusted p-value-filtered DEGs (n = 53) reveals that GSK-J4 treatment not only reverses IMQ-induced upregulation but also enhances expression of genes linked to antigen processing **(**Fig. 6B**)**.

Gene Ontology (GO) analysis of classical monocytes revealed that GSK-J4 treatment significantly downregulated GO terms associated with cytokine-mediated signaling (GO:0001960), TNF-α (GO:0032760) and IL-1β production (GO:0032731), canonical glycolysis (GO:0061621), regulation of monocyte differentiation (GO:0045655), complement receptor-mediated signaling pathway (GO:0002430), and neutrophil chemotaxis (GO:0030593), all of which were prominently upregulated in IMQ-treated skin **(**Fig. 6D**)**. In contrast, gene sets related to antigen presentation via MHC class II (GO:0002503, GO:0019886) and T cell activation (GO:0050870) were positively enriched in the GSK-J4 group relative to IMQ** (**Fig. 6D**)**. Together, these analyses indicate that GSK-J4 treatment substantially remodels the transcriptional state of classical monocytes, reducing inflammatory and metabolic programs while increasing the representation of antigen-presentation-associated transcriptional programs.

Because GSK-J4 treatment increased the representation of CD4⁺FOXP3⁺ Tregs in IMQ-treated mice **(**Fig. 4G**)**, we analyzed Treg gene-expression profiles and visualized the union of significant DEGs (adj. P < 0.05) across the three contrasts in a heatmap. Analysis of significant DEGs across the three experimental groups revealed distinct Treg transcriptional profiles following IMQ exposure and GSK-J4 treatment **(**Fig. 6E**)**. IMQ treatment altered expression of several genes associated with T-cell activation and effector responses, including *Junb*, *Egr2*, and *Tnfrsf9*, consistent with transcriptional adaptation of Tregs to the inflammatory skin environment. Notably, genes such as *Junb* and *Gzmb*, associated with Treg suppressive capacity, were also modestly upregulated in the IMQ condition, possibly reflecting a compensatory regulatory response **(**Fig. 6E**)**. The volcano plot highlights the top 50 significantly altered genes in skin Tregs (BH-adjusted P < 0.05) **(**Fig. 6F**)**. Among the upregulated genes following GSK-J4 treatment were several associated with regulatory functions, including *Itgb8* and *Il10*, while *Traf2* was significantly downregulated, suggesting an induction of anti-inflammatory programs. Additional genes upregulated following GSK-J4 treatment included proliferation-associated factors and regulatory genes such as *Mki67, Ebi3, Ccl5,* and *Socs1* **(**Fig. 6F**)**.

Comparison of Treg cells from IMQ-treated mice to sham controls showed significant enrichment of inflammatory processes, including regulation of type II interferon production (GO:0032649), positive regulation of acute inflammatory response (GO:0002675), and pathways related to CD4^+^ and CD8^+^ alpha–beta T cell differentiation (GO:0043374 and GO:0043367) **(**Fig. 6G**)**. Prominently, treatment with GSK-J4 in IMQ-treated mice led to a reversal of many of these IMQ-induced inflammatory pathways. Specifically, the enrichment of type II interferon (GO:0032649) and IL-6 regulatory pathways (GO:0032715) was markedly suppressed **(**Fig. 6G**)**. Additionally, GSK-J4 treatment attenuated IMQ-induced enrichment of chemokine-mediated signaling (GO:0070098) and TNF response (GO:0071356), further supporting a Treg transcriptional state characterized by reduced representation of inflammatory-response programs. Together, these data demonstrate that KDM6 inhibition via GSK-J4 significantly alters the transcriptional landscape of Tregs in inflamed skin, skewing them away from inflammatory phenotypes and toward a regulatory state.

### GSK-J4 restores H3K27me3 occupancy at the *Il1b* and *Tnf* promoters in inflammatory monocytes

**Fig. 7:**
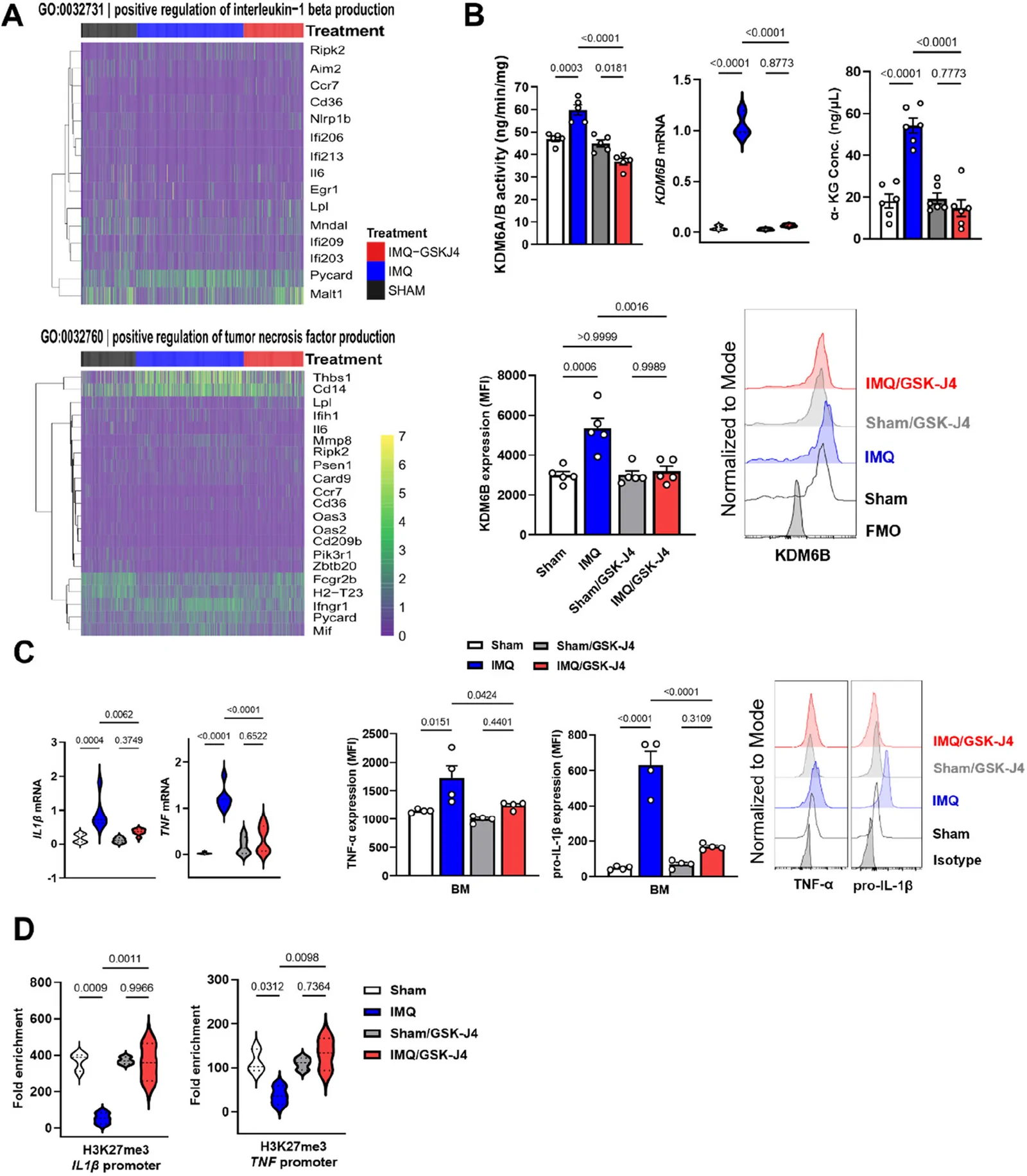
GSK-J4 treatment restores H3K27me3 occupancy at the Il1b and Tnf promoters in inflammatory monocytes. **A** Heatmaps of differentially expressed genes in classical monocytes isolated from skin of sham-, IMQ-, and IMQ/GSK-J4-treated mice. Shown are representative inflammatory genes involved in TNF-α production (GO:0032760) and IL-1β production (GO:0032731) with color scales indicating relative expression levels. The color scale indicates standardized expression levels (z-scores), with yellow representing higher expression and violet representing lower expression relative to the mean. **B** KDM6A/B demethylase activity, *Kdm6b* mRNA expression, KDM6B protein expression, and intracellular α-KG levels in BM monocytes from sham-, IMQ-, sham/GSK-J4-, and IMQ/GSK-J4-treated mice. **C** *Tnf* and *Il1b* mRNA expression measured by RT-qPCR and intracellular TNF-α and pro-IL-1β protein expression measured by flow cytometry in BM monocytes from the indicated treatment groups*.* **D** ChIP-qPCR analysis of H3K27me3 at *Tnf* and *Il1b* promoters in BM monocytes from sham-, IMQ-, GSK-J4-, and IMQ/GSK-J4-treated mice; isotype-matched IgG controls were run in parallel, and signals were normalized to input. Data are representative of three independent experiments (*n* = 4–6 mice per group) and are presented as mean ± SEM. Statistical significance was determined using one-way ANOVA with the Tukey’s multiple comparison test

To further dissect the mechanisms underlying the pathogenic activation of monocytes in IMQ-induced psoriasis, we performed pathway enrichment analysis on differentially expressed genes (DEGs) identified in the classical monocyte populations. Further analysis showed the upregulation of gene sets associated with the regulation of TNF production (GO:0032760) and IL-1β production (GO:0032731) in classical monocytes from the skin of the IMQ-psoriasis mouse model **(**Fig. 7A**)**. BM monocytes were isolated from sham-, IMQ-, sham/GSK-J4-, and IMQ/GSK-J4-treated mice on day 6. IMQ treatment increased KDM6A/B demethylase activity and *Kdm6b* expression in BM monocytes compared with sham-treated controls **(**Fig. 7B**)**. GSK-J4 treatment reduced KDM6A/B activity and *Kdm6b* expression and attenuated the IMQ-associated increase in intracellular α-KG **(**Fig. 7B**)**. These IMQ-associated changes were accompanied by heightened *Il1b* and *Tnf* expression at both the transcriptional and protein levels; these NF-κB-regulated cytokines are important contributors to psoriatic skin inflammation **(**Fig. 7C**)**. Notably, GSK-J4 treatment significantly reduced KDM6A/B activity, lowered *Kdm6b* expression, partially normalized intracellular α-KG, and was accompanied by reduced IL-1β and TNF-α expression, demonstrating its effectiveness in mitigating the inflammatory response **(**Figs. 7A-C**)**.

Mechanistically, KDM6B functions through H3K27me3 demethylation at proinflammatory gene promoters [14, 39]. Reduced H3K27me3 is associated with a more transcriptionally permissive chromatin state at inflammatory loci [38, 40]. We therefore examined H3K27me3 occupancy at selected regulatory regions of the *Il1b* and *Tnf* genes by ChIP-qPCR. H3K27me3 occupancy was reduced at these analyzed regions in BM monocytes from IMQ-treated mice and increased following GSK-J4 treatment **(**Fig. 7D**).** Importantly, GSK-J4 treatment significantly reversed these changes, decreasing KDM6A/B activity, *Kdm6b* expression, IL-1β and TNF-α gene expression, and restoring H3K27me3 levels at NF-κB-binding sites in the *Il1b* and *Tnf* promoter regions. While monocytes are likely the primary targets in this context, we cannot exclude potential effects of GSK-J4 on other immune cell subsets. Together, these results demonstrate that IMQ-induced inflammatory activation of monocytes is associated with increased KDM6A/B activity, increased *Kdm6b* expression, reduced H3K27me3 occupancy at regulatory regions of the *Il1b* and *Tnf* genes, and increased expression of these inflammatory mediators. Pharmacological KDM6B inhibition with GSK-J4 reverses these changes, linking KDM6-associated H3K27me3 remodeling to the inflammatory transcriptional state of monocytes during IMQ-induced psoriasis-like inflammation.

### GSK-J4 attenuates inflammatory metabolic remodeling of monocytes in the IMQ-induced psoriasis model

**Fig. 8:**
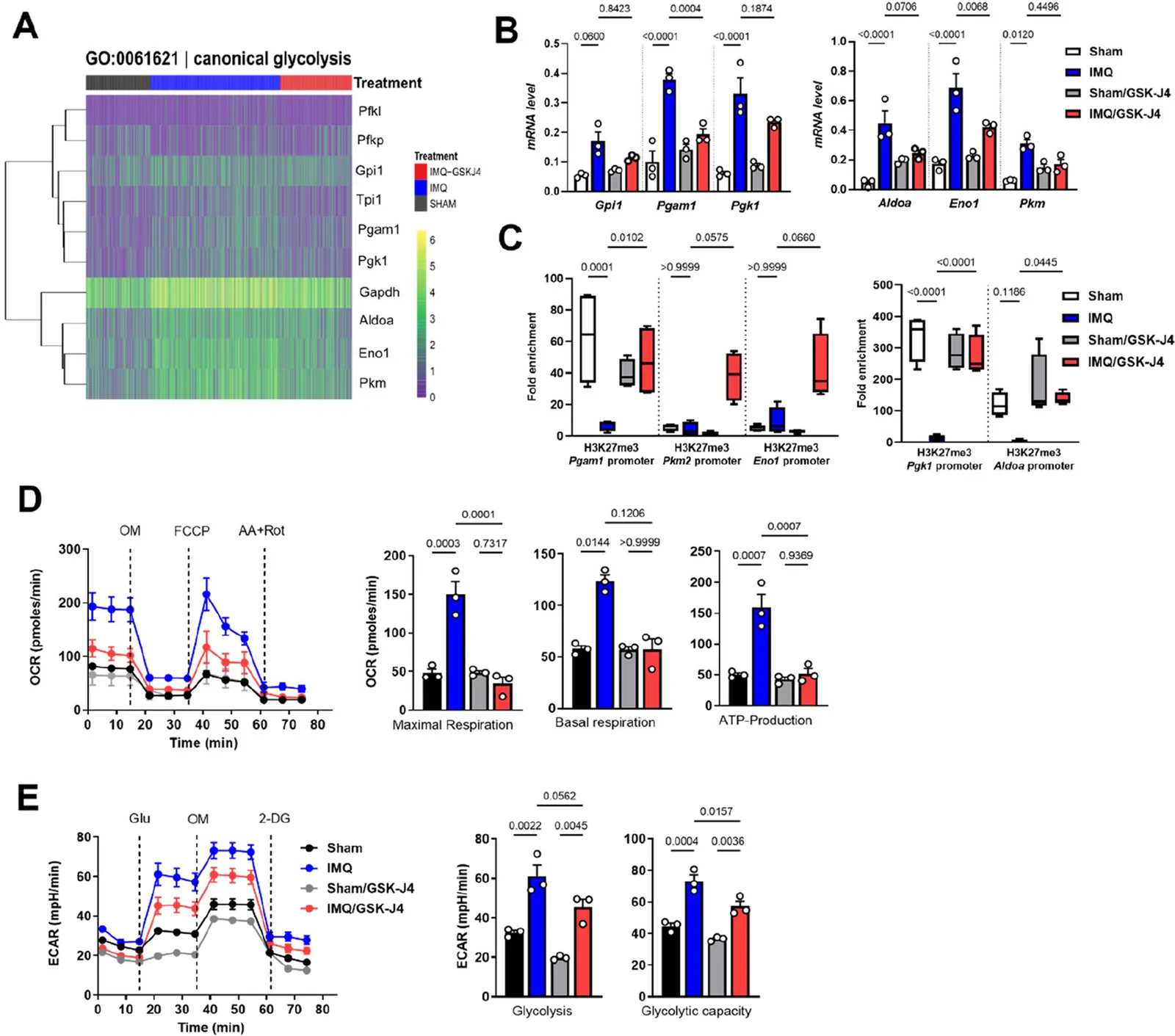
Metabolic remodeling of inflammatory monocytes in the IMQ-induced psoriasis-like mouse model. **A** Heatmap of expression of genes encoding canonical glycolytic enzymes in classical skin monocytes from IMQ-treated mice compared with sham- and IMQ/GSK-J4-treated mice. The color scale indicates standardized expression levels (z-scores), with yellow representing higher expression and violet representing lower expression relative to the mean. **B** Gene expression levels of key glycolytic enzymes in BM monocytes, assessed by RT-qPCR. **C** ChIP-qPCR analysis of H3K27me3 enrichment at the regulatory regions of glycolytic genes in BM monocytes from IMQ-treated mice with and without GSK-J4 compared to sham-treated mice. Isotype-matched IgG controls were analyzed in parallel, and ChIP signals were normalized to input chromatin. **D** Seahorse analysis of cellular OCR, basal respiration, maximal respiration, and ATP production. OM: oligomycin; FCCP: carbonyl cyanide-4-(trifluoromethoxy)phenylhydrazone (uncoupler); AA + Rot: antimycin A + rotenone. **E** Seahorse analysis of ECAR, glycolysis, and glycolytic capacity in BM monocytes from IMQ-treated mice with and without GSK-J4 compared to sham-treated mice with sequential injections of glucose (Glu), oligomycin (OM), and 2-deoxy-D-glucose (2-DG). Data are representative of three independent experiments (*n* = 4–6 mice per group) and are presented as mean ± SEM. Statistical significance was determined using one-way ANOVA with the Tukey’s multiple comparison test

Recent studies indicate that reprogramming of cellular metabolism and epigenetic modifications can regulate monocyte phenotype and function [12, 41]; however, little is known about the metabolic programs that characterize monocytes in IMQ-psoriasis. GO analysis identified canonical glycolysis among the most significantly enriched pathways in IMQ-treated versus sham-treated classical monocytes (Fig. 8A**)**. Specifically, 10 out of 18 canonical glycolysis-related genes (GO:0061621), including *Pkm, Eno1, Aldoa, Gapdh, Pgk1, Pgam1, Tpi1, Gpi1, Pfkp, and Pfk1,* were upregulated in classical skin monocytes from IMQ-treated mice compared to sham controls **(**Fig. 8A**)**. Additionally, the gene expression of these glycolytic enzymes was significantly increased in BM monocytes of IMQ-treated mice compared to sham-treated controls **(**Fig. 8B**)**. Mice treated with IMQ in combination with GSK-J4 displayed a significant reduction in the expression of canonical glycolytic enzyme genes, including *Pgam1*, *Pgk1*, *Aldoa*, *Pkm*, and *Eno1*, in BM monocytes **(**Fig. 8B**)**. We next investigated whether changes in glycolytic gene expression were associated with altered H3K27me3 occupancy at their regulatory regions. ChIP-qPCR analysis demonstrated reduced H3K27me3 occupancy at the analyzed regulatory regions of *Pgam1*, *Pgk1*, and *Aldoa* in BM monocytes from IMQ-treated mice compared with sham controls **(**Fig. 8C**)**. GSK-J4 treatment increased H3K27me3 occupancy at these regions and also altered H3K27me3 occupancy at the analyzed *Pkm2* and *Eno1* regions **(**Fig. 8C**)**. Thus, increased expression of glycolysis-associated genes during IMQ-induced inflammation is accompanied by reduced occupancy of the repressive H3K27me3 histone modification at selected glycolytic loci, and these epigenetic changes are reversed or attenuated by GSK-J4 treatment.

Inflammatory monocyte activation is accompanied by substantial metabolic remodeling with enhanced glycolysis, while mitochondrial responses vary with context and activation state [12, 42]. To systematically characterize the metabolic activity of monocytes from IMQ-treated mice, we measured the oxygen consumption rate (OCR) and extracellular acidification rate (ECAR) in BM monocytes, indicative of oxidative phosphorylation (OXPHOS) and glycolytic activity, respectively. Consistent with observations in monocytes from psoriasis patients [17] and the enrichment of canonical glycolysis-related genes **(**Fig. 8A**)**, IMQ-treated monocytes displayed elevated basal and maximal OCR as well as spare respiratory capacity, reflecting enhanced mitochondrial respiration. Treatment with GSK-J4 significantly reduced OCR parameters, consistent with reduced mitochondrial respiratory activity **(**Fig. 8D**)**. Furthermore, IMQ-treated BM monocytes exhibited increased ECAR, indicative of heightened glycolytic activity and glycolytic capacity, both of which were significantly attenuated by GSK-J4 treatment **(**Fig. 8E**)**. Collectively, these findings demonstrate that IMQ-induced inflammatory activation is associated with coordinated metabolic remodeling of BM monocytes, characterized by increased mitochondrial respiration and glycolytic activity. GSK-J4 attenuates both metabolic programs and restores H3K27me3 occupancy at selected glycolysis-associated loci, linking GSK-J4-sensitive KDM6-associated chromatin remodeling to the metabolic state of inflammatory monocytes.

## Discussion

Our study identifies KDM6B-associated epigenetic and metabolic remodeling as an important feature of inflammatory monocyte activation during psoriasis-like inflammation. Peripheral depletion of classical Ly6C^hi^ monocytes or GSK-J4 treatment reduced both skin and systemic inflammation in the IMQ-psoriasis mouse model, even when treatment began after disease onset. Increased KDM6B expression and KDM6A/B activity were associated with reduced H3K27me3 occupancy at NF-κB-responsive *Il1b* and *Tnf* promoters and key glycolytic genes (*Pgam1, Pgk1,* and *Aldoa*), linking chromatin opening to enhanced glycolytic flux and elevated inflammatory cytokine production. Conversely, GSK-J4 restores H3K27me3, suppresses both the metabolic and inflammatory pathways, and normalizes monocyte metabolism.

Previous work suggested that psoriasis is a disease primarily driven by keratinocyte-T cell crosstalk, particularly the IL-23/IL-17 axis [1, 4, 43]. However, increasing evidence implicates innate myeloid populations as important amplifiers of psoriatic inflammation. Our data show an association between monocyte accumulation and IMQ-induced inflammation: monocytes infiltrate lesional skin in large numbers, adopt a transcriptional program dominated by NF-κB, and show increased expression of TNF-α and pro-IL-1β that is sensitive to GSK-J4 treatment. The concurrent increase in α-KG raises the possibility that the metabolic state of inflammatory monocytes contributes to KDM6-family demethylase activity. Together with the observed changes in H3K27me3 occupancy and glycolytic gene expression, these findings suggest a potential interaction between metabolic and epigenetic remodeling. Establishing a causal feed-forward mechanism will require independent perturbation of metabolic intermediates and KDM6B-specific genetic approaches. Blocking KDM6B breaks this loop, limits neutrophil recruitment, and is accompanied by an increase in FOXP3⁺ Treg cell numbers, implying that monocyte reprogramming has downstream effects on adaptive immunity.

The anti-inflammatory activity of GSK-J4 was also observed in the IL-36α/IL-17A-driven model, providing evidence that its effects are not restricted to IMQ-induced inflammation. The attenuation of inflammatory responses across these mechanistically distinct models strengthens the evidence that KDM6B-associated processes participate in psoriasis-like inflammation downstream of multiple inflammatory stimuli. Nevertheless, both models represent experimentally induced murine inflammation and cannot fully reproduce the chronicity and cellular complexity of human psoriasis.

Recent studies show that KDM6B drives maladaptive myeloid reprogramming across multiple inflammatory diseases [18, 44–46]. *Davis *et al. identified KDM6B as a central regulator of monocytes/macrophage-driven inflammation in the development of abdominal aortic aneurysms (AAAs) [14]. Both human AAA specimens and mouse models showed markedly increased KDM6B expression in monocytes and macrophages infiltrating the aortic wall [14]. In diabetic wounds, excessive KDM6A/B activity impairs reparative monocyte and macrophage function through aberrant activation of the cGAS-STING pathway [39], whereas in glioblastoma, myeloid-specific *Kdm6b* deletion or pharmacological inhibition with GSK-J4 restores antigen presentation, enhances type I interferon signaling, and sensitizes tumors to anti-PD-1 therapy [13].

Mechanistically, KDM6B promotes inflammation by removing the repressive H3K27me3 mark on promoters of NF-κB target genes such as *Il1b, Il12,* and *Tnf*, thereby allowing their transcription. Upstream, type I interferon (IFN-β) induces KDM6B expression via the JAK1/STAT1 pathway [14]. Audu et al. further showed that KDM6B acts as an epigenetic ‘gatekeeper’, deciding whether wound-resident macrophages resolve or prolong inflammation. During normal repair, a transient IFN-β increase activates JAK1/3-STAT3 signaling early after injury, producing a short-term increase in KDM6B. In obesity-associated diabetic wounds, however, persistent IL-6 maintains the same pathway, generating a delayed but sustained KDM6B induction that removes H3K27me3 from *Il1b, Tnf,* and the cGAS-STING gene *Tmem173* and amplifies NF-κB signaling [39]. In systemic SLE monocytes, chronic type I IFN exposure elevates intracellular α-KG, boosts KDM6A/B activity, reduces H3K27me3 at interferon-stimulated gene promoters and sustains an inflammatory “trained-immunity” state [18]. These findings share features with epigenetic-metabolic programs described in trained immunity; however, the present study did not directly test innate immune memory. Consistent with findings in SLE [18], GSK-J4 treatment in the IMQ model of psoriasis restores repressive H3K27me3 and dampens pathogenic transcriptional programs, emphasizing the translational potential of targeting KDM6B in autoinflammatory disease.

Despite its efficacy, GSK-J4 lacks absolute selectivity and can inhibit non-histone proteins and the related demethylase KDM6A at higher concentrations [16, 46]**.** Moreover, the specific contribution of monocytes to psoriasis-induced systemic inflammation remains to be defined. Systemic KDM6B inhibition is likely to impact multiple immune and non-immune lineages, including Th17 cells, dendritic cells, and keratinocytes. Since earlier studies showed that KDM6B inhibition prevents Th17 differentiation, a cell-type-specific *Kdm6b*-deficient mouse would be invaluable for examining these lineage-specific effects [37, 47]. A further limitation is.

that the IMQ-psoriasis mouse model reflects only an acute inflammatory response, highlighting the need to investigate chronic psoriasis models as well.

Despite the success of IL-17 and IL-23 biologic therapies, up to one-third of patients remain non-responders or eventually relapse [48]. Future studies should determine whether KDM6-targeted approaches can complement existing cytokine-directed therapies. Emerging modalities such as locus-selective protein degraders and allosteric inhibitors promise greater specificity than GSK-J4 while retaining comparable anti-inflammatory efficacy [16].

Collectively, our findings support KDM6B-associated H3K27me3 remodeling as a contributor to inflammatory and metabolic reprogramming in monocytes during psoriasis-like inflammation. Pharmacological KDM6B inhibition with GSK-J4 modulated both innate and adaptive immune-cell responses, supporting further investigation of this pathway as a therapeutic target.

## Supplementary Information

Supplementary Material 1.

## Data Availability

The raw files and raw count table from the single-cell RNA sequencing data can be accessed at Gene Expression Omnibus (GEO) with accession number GSE312272. The dataset reported in this work can be explored under https://shiny.imbei.uni-mainz.de/iSEE_KDM6B_inhibition/ with an interactive dashboard based on the iSEE package.
